# The Relationship Between Echocardiographic Calcification Score and Grade of Knee Osteoarthritis

**DOI:** 10.7759/cureus.48869

**Published:** 2023-11-15

**Authors:** Ahmet Kivrak, Alp Yildirim, Levent Horoz, Galip Beltir

**Affiliations:** 1 Cardiology, Ankara Etlik City Hospital, Ankara, TUR; 2 Cardiology, Kirsehir Training and Research Hospital, Kirsehir, TUR; 3 Orthopaedics and Traumatology, Kirsehir Training and Research Hospital, Kirsehir, TUR

**Keywords:** osteoarthritis, joint disease, echocardiography, cardiac disease, calcification

## Abstract

Background and objective

It has been suggested that knee osteoarthritis (KOA) is associated with the development of calcification and an increased risk of cardiovascular (CV) disease, while the contribution of KOA grade is not clearly known enough. This study aimed to investigate the relationship between the grade of KOA, the echocardiographic calcification score (echo-CCS), and CV risk assessment.

Methods

This cross-sectional study involved 204 patients diagnosed with KOA and classified according to the Kellgren-Lawrence staging criteria. Echo-CCS was obtained according to the presence of calcification in the aortic valve, aortic root, mitral ring, papillary muscle and ventricular septum. Framingham risk score (FRS) was used for CV risk assessment.

Results

Calcification was detected in 79.4% of patients*. *The median FRS, echo-CCS, and high-sensitivity C-reactive protein (hs-CRP) levels increased as the KOA grade increased (p<0.05). A one-grade increase in KOA increased the odds of echo-CCS 1-2 group by 5.15 fold (vs. no calcification group) (OR=5.15, p=0.003), while it increased the odds of echo-CCS ≥3 group by 4.61 fold (vs. echo-CCS 1-2 group) (OR=4.61, p=0.003). Median echo-CSS and hs-CRP were higher in the high CV risk group than in the moderate and low CV risk groups.

Conclusion

The majority of patients with KOA had intracardiac calcification. An increased KOA grade was associated with higher echo-CSS and FRS. These findings indicate that patients with higher grades of KOA may be predisposed to developing subclinical atherosclerosis.

## Introduction

Osteoarthritis (OA) is the most common joint disease associated with several heterogeneous factors, characterized by gradual loss of cartilage and changes in the joint margins [1]. OA, a progressive disease, is accompanied by various comorbidities in the majority of patients [2]. Comorbidities due to insulin resistance or high blood pressure, which cause the activation of the inflammatory pathway, may increase cartilage damage, compromise the repair process, and increase the risk of cardiovascular (CV) events [3,4].

Increasing evidence has indicated that patients with OA tend to develop calcifications and CV disease [5-7]. A large population study has reported a positive association between incident arterial calcification and incident OA [8]. Calcifications evaluated on echocardiography are an important prognostic factor for coronary artery disease (CAD) and future CV events [9]. The echocardiographic calcification score (echo-CCS) has been an independent predictor for atherosclerosis, and it is associated with increased all-cause mortality in patients at high CV risk [9,10]. Very few studies have specifically investigated the association between knee OA (KOA) and echo-CCS [11]. However, the relationship between intracardiac calcification and the severity of KOA remains insufficiently defined.

It has been suggested that KOA plays a role in the etiopathogenesis of atherosclerosis, with a common link even in low-grade inflammation [12]. In this context, we hypothesized that a majority of patients with KOA may have calcification in the cardiac structures and high levels of echo-CSS, an independent predictor of atherosclerosis. This study aimed to investigate the relationship between the grade of KOA, the echo-CCS, and CV risk assessment.

## Materials and methods

This cross-sectional study was conducted between March 2021 and May 2021 in the Kırşehir Training and Research Hospital Cardiology and Orthopedics departments. All procedures followed were in accordance with ethical principles and Declaration Helsinki (as revised in Brazil 2013). The local ethics committee approved the study protocol (Date/No: 04.2021/E-42884709-020). Written informed consent was obtained from all participating patients.

The sample size was determined using Cochran's formula [13]: n = (p × [1 - p] × Z_α_²) / d², where Z_α_ = 1.96 at a 95% confidence level. Here, "p" represents the expected prevalence or proportion, and "d" stands for the desired precision. In previous studies, the prevalence of KOA has been reported to be between 15-34%​​​ [14,15]. In this context, assuming a mean prevalence of 25% for KOA and a precision of 10%, it has been determined that a sample size of at least 73 patients is required with a 95% confidence level and 80% power.

Study population

A total of 204 patients who applied to the Training and Research Hospital Orthopedics Outpatient Clinic and were diagnosed with KOA were included in the study. The diagnosis of KOA was established following the American College of Rheumatology (ACR) criteria, which involve knee pain and the requirement for a minimum of three of the following criteria: age over 50 years, stiffness lasting less than 30 minutes, crepitus, bony tenderness, bony enlargement, and the absence of palpable warmth. Additionally, routine radiographic examinations were conducted to confirm the diagnosis of KOA [16]. The Kellegren-Lawrence staging criteria were then utilized to grade the severity of KOA based on the radiographic findings [17].

Patients using drugs (thiazide, lithium, estrogen, etc.) to increase serum calcium and phosphorus levels, immobilized, fragile patients, patients with diseases that cause hypercalcemia (primary hyperparathyroidism, malignancies, granulomatous diseases), uncontrolled hypertension, a history of CAD, chronic kidney disease, and congestive heart disease were excluded.

All patients' detailed medical histories were taken, physical examinations were performed, and baseline characteristics were recorded. Hypertension was defined as having repeated office blood pressure measurements of ≥140/90 mmHg, in line with the 2018 European Society of Cardiology and the European Society of Hypertension for the management of arterial hypertension [18], or using antihypertensive drugs. After hospital admission, all patients were rested for five minutes, followed by three different blood pressure measurements at five-minute intervals, and the averages were taken. Diabetes mellitus was defined as having multiple measurements of fasting plasma glucose levels of ≥ 126 mg/dL, hemoglobin A1C of ≥6.5, random plasma glucose ≥200 mg/dL [19], or currently using antidiabetic drugs.

Laboratory measurements

Fasting blood samples of all patients were taken on an empty stomach in the morning before transthoracic echocardiography examination. Laboratory parameters, including complete blood count and lipid panel, were measured using Beckman Coulter LH 780 (Mervue, Galway, Ireland). Thus, the levels of hemoglobin (photometrically), platelets (impedance method), high-sensitivity C-reactive protein (hs-CRP) (immunoturbidimetric method) (normal range: 0.15-5 mg/L), albumin (bromine cresol green method), triglycerides and total cholesterol (enzymatic colorimetric method) and high-density lipoprotein (HDL; homogeneous enzymatic colorimetric method) were determined. Low-density lipoprotein (LDL) levels were calculated using the Friedewald formula [20]. 

Transthoracic echocardiographic assessment

All transthoracic echocardiographic evaluation was performed by a cardiologist blind to patient information using the GE Vivid S6 Echo device (General Electric Medical Systems, Milwaukee, WI, USA). Parasternal long and short-axis views and apical four, two-chamber, and long-axis views were recorded in compliance with the recommendations of the European Association of CV Imaging [21]. The Biplane Simpson Method calculated left ventricular ejection fraction (LVEF).

**Figure 1:**
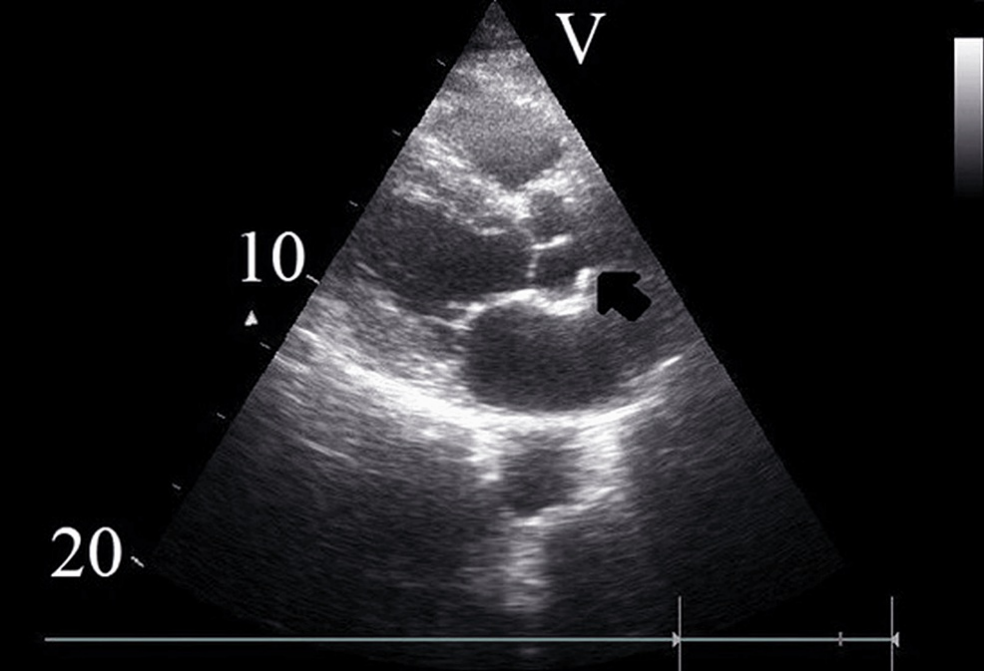
Evaluation of calcification in the aortic root by echocardiography in patients with knee osteoarthritis.

Cardiac structures of the aortic valve, aortic root (Figure 1), mitral ring, papillary muscles and ventricular septum were evaluated for the presence or absence of calcification. Later, these values were collected, and the echocardiographic calcification scores were calculated to be between 0-5 [9]. Patients were classified according to their calcification score as follows: 0 (no calcification), echo-CSS 1-2 and echo-CSS >3. 

Assessment of the CV risk

The Framingham risk score (FRS), which includes age, gender, total cholesterol, HDL, systolic blood pressure, and smoking habit, was used to assess CV disease risk. Ten-year risk percentage was derived from the total score. Accordingly, absolute risk of CV disease was classified as low risk (<10%), medium risk (10-20%), and high risk (>20%) [22].

Statistical analysis 

Statistical analysis of the study was performed using IBM SPSS v25 software for Windows (IBM Corp., Armonk, NY, USA). The normality assumption for quantitative variables was tested with Shapiro-Wilk tests. Data are given as mean ± standard deviation or median (quartiles of 25-75th) relative to the normal distribution for numerical variables and n (%) for categorical variables. Chi-square and Fisher exact tests were used to compare categorical data. ANOVA (post hoc: Bonferroni test) or Kruskal-Wallis H test (post hoc: Dunn’s test) were used to compare numerical variables between the groups according to the normality distribution. Potential risk factors for echo-CSS were determined by multinomial logistic regression analysis. Statistical significance (*) was accepted as p < 0.05.

## Results

The mean age of 204 patients included in the analysis was 63.4±7.4 years (range = 51-85 years) and they were mostly female (86.3%). Grade III and Grade IV rates were 23.5% and 16.7%, respectively. The median echo-CCS was 2 (range = 0-5) and the rate of patients with no calcification was 20.6% and the rate of patients with calcification in single structure was 26.5%. The baseline characteristics are listed in Table 1.

**Table 1:** Baseline characteristics of patients with knee osteoarthritis Numerical variables were presented as mean ± SD or median (IQR), and categorical variables as numbers (%). * is indicated statistical significance. For post-hoc test: ^a^ *vs.* Grade I. ^b^ *vs.* Grade II. ^c^ *vs.* Grade III. ^d^ *vs.* Grade IV. AVC, aortic valve calcification; ARC, aortic root calcification; BMI, Body mass index; Echo-CCS, echocardiographic calcification score; FRS, Framingham risk score; HDL-C, high density lipid cholesterol; hs-CRP, high-sensitivity C reactive protein; LDL-C, low density lipid cholesterol; LVEF, left ventricle ejection fraction; MAC, mitral annulus calcification; PMC, papillary muscle calcification; SC, septum calcification; Total-C, total cholesterol; WBC, white blood cell.

Variables	All population n = 204	Grade of knee osteoarthritis				p
		Grade I n = 73	Grade II n = 49	Grade III n = 48	Grade IV n = 34	
Age, years	63.4±7.4	60.6±6.6	61.1±6.3	65.7±4.4^ab^	66.8±5.3^ab^	<0.001*
Gender, n (%)						
Female	176 (86.3)	65 (89.0)	46 (93.9)	38 (79.2)	27 (79.4)	0.090
Male	28 (13.7)	8 (11.0)	3 (6.1)	10 (20.8)	7 (20.6)	
BMI, kg/m^2^	26.8±2.4	26.4±2.1	26.7±2.4	27.2±2.8	27.7±2.2	0.150
Smoking, n (%)	40 (19.6)	10 (13.7)	5 (10.2)	3 (6.3)	12 (35.3)	0.004*
Hypertension, n (%)	120 (58.8)	20 (27.4)	30 (61.2)	41 (85.4)	29 (85.3)	<0.001*
Diabetes mellitus, n (%)	68 (33.3)	3 (4.1)	16 (32.7)	24 (50.0)	27 (79.4)	<0.001*
FRS	12.3 (7.7-22.0)	7.0 (5.3-8.0)^bcd^	13.7 (12.0-15.7)^acd^	19.2 (17.2-24.3)^abd^	24.8 (21.6-28.5)^abc^	<0.001*
Low, n (%)	79 (38.7)	71 (97.3)	1 (2.0)	5 (10.4)	2 (5.9)	<0.001*
Moderate, n (%)	65 (31.9)	2 (2.7)	45 (91.8)	16 (33.3)	2 (5.9)	
High, n (%)	60 (29.4)	0	3 (6.1)	27 (56.3)	30 (88.2)	
SBP, mmHg	135.1±15.2	124.4±11.7^bcd^	138.0±9.6^ad^	141.0±14.1^ad^	146.0±16.7^abc^	<0.001*
DBP, mmHg	86.6±8.7	73.0±9.3^bcd^	78.4±6.4	78.5±9.1	79.1±7.5	<0.001*
LVEF, %	64.0±3.0	65.4±2.7^bcd^	64.0±2.3	63.7±2.1	63.0±1.9	0.027*
Echo CCS	2 (1-3)	1 (0-1)^bcd^	2 (1-3)^acd^	3 (2-3)^abd^	4 (3-5)^abc^	<0.001*
0, n (%)	42 (20.6)	35 (47.9)	7 (14.3)	0	0	<0.001*
1-2, n (%)	87 (42.6)	37 (50.7)	29 (59.2)	18 (37.5)	3 (8.8)	
≥3, n (%)	75 (36.8)	1 (1.4)	13 (26.5)	30 (62.5)	31 (91.2)	
PMC, n (%)	45 (22.1)	2 (2.7)	7 (14.3)	17 (35.4)	19 (55.9)	<0.001*
MAC, n (%)	65 (31.9)	4 (5.5)	11 (22.4)	20 (41.7)	30 (88.2)	<0.001*
AVC, n (%)	129 (63.2)	24 (32.9)	34 (69.4)	41 (85.4)	30 (88.2)	<0.001*
ARC, n (%)	94 (46.1)	3 (4.1)	22 (44.9)	40 (83.3)	29 (85.3)	<0.001*
SC, n (%)	41 (20.1)	10 (13.7)	4 (8.2)	11 (22.9)	16 (47.1)	<0.001*
WBC, ×10^3^/µL	8.3±2.3	8.3±2.4	8.5±2.5	8.4±2.4	8.8±1.6	0.746
Platelet, ×10^3^/µL	274.1±61.8	251.4±54.1	268.4±50.6	294.0±69.8^ab^	302.7±61.6^ab^	<0.001*
Hemoglobin, g/dL	14.4±2.3	14.7±1.2	14.3±1.4	14.5±3.6	13.8±2.7	0.341
Glucose, mg/dL	113.5 (99.0-140.0)	101.0 (80.0-118.0)	106.0 (88.0-123.0)	120.0 (90.0-141.0)^ab^	145.0 (95.0-188.0)^ab^	<0.001*
hs-CRP, mg/L	0.9 (0.5-2.6)	0.5 (0.2-0.8)^bcd^	1.0 (0.5-1.3)^acd^	2.4 (0.7-4.4)^abd^	4.2 (2.2-5.3)^bcd^	<0.001*
Total-C, mg/dL	215.4±51.8	203.1±41.1	217.7±58.8	218.0±44.3	245.8±58.2^bcd^	<0.001*
HDL-C, mg/dL	53.4±10.2	53.6±12.0	55.8±11.0	51.7±7.3	52.0±8.0	0.177
LDL-C, mg/dL	129.3±40.0	115.2±34.5^bcd^	132.2±36.9	135.4±37.1	151.2±31.2^bcd^	<0.001*

**Figure 2:**
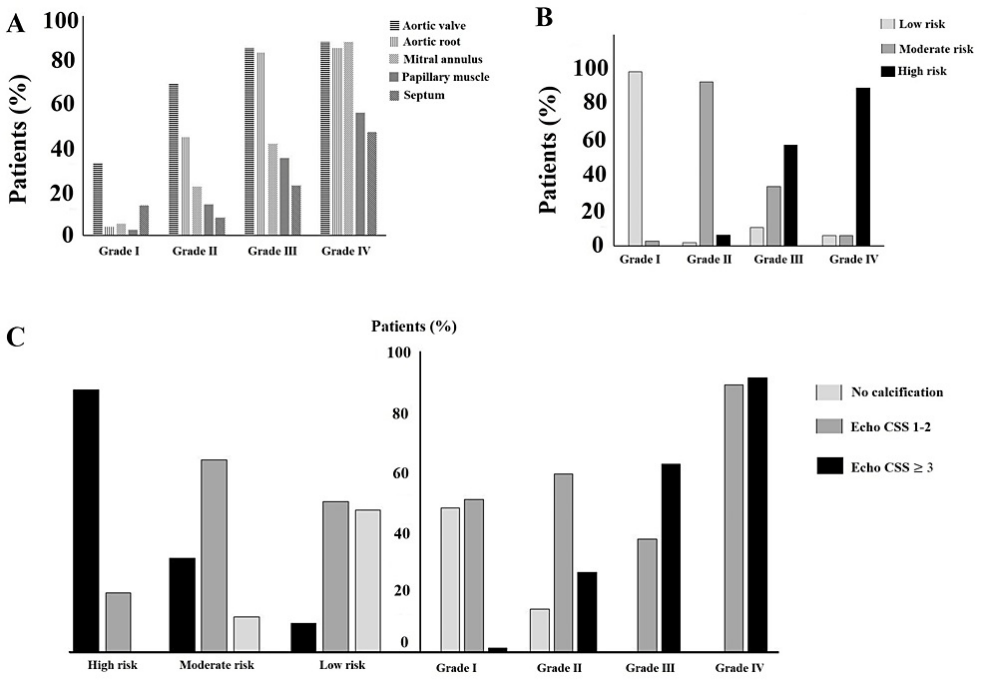
Distribution of calcification findings in cardiac structures by grade of knee osteoarthritis (A). A 10-year cardiovascular risk classification by grade of knee osteoarthritis (B). Distribution of echo-CSS by grade of knee osteoarthritis and 10-year cardiovascular risk classifications (C). Echo-CSS, echocardiographic calcification score

The mean age and comorbidity rates were higher in the Grade III-IV group than Grade I-II groups. Calcification rates in cardiac structures according to grade of KOA are presented in Figure 2A. The median echo-CSS and FRS levels were increased with the KOA grade (Table 1). The distribution of the 10-year CV risk classification by KOA grade is shown in Figure 2B. While the median FRS score was lower in the group without calcification, it was higher in the echo-CSS ≥3 group (echo-CSS 0: 7.3 *vs.* echo-CSS 1-2: 11.7 *vs.* echo-CSS ≥3: 24.0, p<0.001) (Table 2). Echo-CCS levels showed a positive correlation with KOA severity (r = 0.754, p<0.001) and FRS (r = 0.713, p<0.001).

**Table 2:** Relationship between clinical findings and echo-CSS Numerical variables were presented as mean ± SD or median (IQR), and categorical variables as numbers (%). * is indicated statistical significance. For the post-hoc test: ^a^ *vs.* echo-CSS 0. ^b^ *vs.* echo-CSS 1-2. ^c^ *vs*. echo-CSS ≥3. AVC, aortic valve calcification; ARC, aortic root calcification; BMI, Body mass index; Echo-CCS, echocardiographic calcification score; FRS, Framingham risk score; HDL-C, high density lipid cholesterol; hs-CRP, high-sensitivity C reactive protein; KOA, knee osteoarthritis; LDL-C, low density lipid cholesterol; LVEF, left ventricle ejection fraction; MAC, mitral annulus calcification; PMC, papillary muscle calcification; SC, septum calcification; Total-C, total cholesterol; WBC, white blood cell.

Variables	Echo CCS			p
	0 n=42	1-2 n=87	≥3 n=75	
Age, years	59.7±5.3	62.7±7.1	66.2±7.9^ab^	<0.001*
Gender, n (%)				
Female	37 (88.1)	78 (89.7)	61 (81.3)	0.299
Male	5 (11.9)	9 (10.3)	14 (18.7)	
BMI, kg/m^2^	26.6±2.1	26.7±2.4	27.3±2.6	0.206
Smoking, n (%)	6 (14.3)	12 (13.8)	22 (29.3)	0.034*
Hypertension, n (%)	13 (31.0)	45 (51.7)	62 (82.7)	<0.001*
Diabetes mellitus, n (%)	5 (11.9)	24 (27.6)	41 (54.7)	<0.001*
Grade of KOA, n (%)				
I	35 (83.3)	37 (42.5)	1 (1.3)	<0.001*
II	7 (16.7)	29 (33.3)	13 (17.3)	
III	0	18 (20.7)	30 (40.0)	
IV	0	3 (3.4)	31 (41.3)	
FRS	7.3 (6.0-8.2)^bc^	11.7 (7.3-16.0)^ac^	24.0 (18.5-25.3)^ab^	<0.001*
Low, n (%)	35 (83.3)	37 (42.5)	7 (9.3)	<0.001*
Moderate, n (%)	7 (16.7)	39 (44.8)	19 (25.3)	
High, n (%)	0	11 (12.6)	49 (65.3)	
SBP, mmHg	129.7±12.2	130.2±14.3	143.8±13.9^ab^	<0.001*
DBP, mmHg	84.0±9.3	85.7±8.9	92.4±7.6^ab^	0.001*
LVEF, %	65.1±3.1^bc^	63.8±3.2	63.6±2.8	0.006*
PMC, n (%)	0	6 (6.9)	39 (52.0)	<0.001*
MAC, n (%)	0	16 (18.4)	49 (65.3)	<0.001*
AVC, n (%)	0	63 (72.4)	66 (88.0)	<0.001*
ARC, n (%)	0	24 (27.6)	70 (93.3)	<0.001*
SC, n (%)	0	11 (12.6)	30 (40.0)	<0.001*
WBC, ×10^3^/µL	7.8±2.1	8.0±2.3	8.6±2.7^ab^	0.015*
Platelet, ×10^3^/µL	263.7±60.2	254.5±49.3	302.5±65.5^ab^	<0.001*
Hemoglobin, g/dL	14.7±1.5	14.4±1.2	14.2±3.4	0.437
Glucose, mg/dL	115.0 (91.5-147.8)	114 (99.0-140.0)	112 (102.0-138.0)	0.642
hs-CRP, mg/L	0.5 (0.3-0.8)^bc^	0.8 (0.4-1.2)^ac^	3.2 (1.0-5.0)^ab^	<0.001*
Total-C, mg/dL	203.4±42.3	209.3±50.7	229.1±55.3^ab^	0.012*
HDL-C, mg/dL	55.2±12.9	52.6±10.4	53.3±8.1	0.393
LDL-C, mg/dL	118.0±30.5	125.2±42.0	140.4±40.0^ab^	0.006*

Grade of KOA and hs-CRP level were common independent risk factors for both echo-CCS 1-2 (vs. no calcification) and echo-CCS ≥3 (vs. echo-CCS 1-2). Accordingly, a one-grade increase in KOA increased the odds of echo-CCS 1-2 by 5.24 fold than without calcification group (OR=5.24, p=0.002), while it increased the odds of echo-CCS ≥3 by 4.96 fold than echo-CCS 1-2 group (OR=4.96, p<0.001) (Table 3).

**Table 3:** Independent factors associated with echo-CSS levels The effects of age, BMI, and comorbid conditions were adjusted in the multivariable regression analysis. Nagelkerke R^2^ = 0.438, p <0.001 for Echo-CCS 1-2 (ref: 0). Nagelkerke R^2^ = 0.648, p <0.001 for Echo-CCS ≥3 (ref: 1-2). * is indicated statistical significance. BMI, Body mass index; CI, confidence interval; Echo-CCS, echocardiographic calcification score; hs-CRP, high-sensitivity C reactive protein; KOA, knee osteoarthritis; LDL-C, low density lipid cholesterol; LVEF, left ventricle ejection fraction; OR, odds ratio; Total-C, total cholesterol; WBC, white blood cell.

Variables	Univariable Regression		p	Multivariable Regression		p
	OR	95% CI		OR	95% CI	
Echo-CCS 1-2 (ref: 0)						
Age	1.08	0.98-1.15	0.089	-	-	-
BMI	1.01	0.85-1.19	0.917	-	-	-
Smoking	0.96	0.33-2.76	0.940	-	-	-
Hypertension	2.39	1.10-5.20	0.028*	-	-	-
Diabetes mellitus	2.82	1.01-8.02	0.050*	-	-	-
Grade of KOA	5.23	2.32-11.78	<0.001*	5.24	1.83-14.91	0.002*
LVEF	0.81	0.68-0.95	0.022*	-	-	-
hs-CRP	2.12	1.14-3.97	0.010*	1.98	1.02-3.86	0.044*
Echo-CCS ≥3 (ref: 1-2)						
Age	1.07	1.02-1.11	0.004*	-	-	-
BMI	1.11	0.97-1.26	0.123	-	-	-
Smoking	2.59	1.18-5.70	0.018*	-	-	-
Hypertension	3.73	1.85-7.56	<0.001*	-	-	-
Diabetes mellitus	3.17	1.65-6.09	0.001*	-	-	-
Grade of KOA	5.52	3.34-9.11	<0.001*	4.96	2.54-9.67	<0.001*
LVEF	0.96	0.83-1.11	0.573	-	-	-
WBC	1.16	1.01-1.35	0.042*	-	-	-
Platelet	1.02	1.01-1.03	<0.001*	1.02	1.01-1.03	0.042*
hs-CRP	1.91	1.51-2.41	<0.001*	1.41	1.02-1.98	0.048*
Total-C	1.04	1.01-1.08	0.021*	-	-	-
LDL-C	1.02	1.01-1.03	0.012*	-	-	-

Median echo-CSS was higher in the high-risk group than moderate and low-risk groups (Low risk: 1 vs. Moderate risk: 2 vs. High risk: 3, p<0.001). Distribution of echo-CSS by 10-year CV risk classifications is shown in Figure 2C. An increase in platelet, hs-CRP and LDL-C levels were associated with the high-risk group (Table 4).

**Table 4:** Parameters associated to the 10-year cardiovascular risk by Framingham risk score in patients with KOA. Numerical variables were presented as mean ± SD or median (IQR), and categorical variables as numbers (%). * is indicated statistical significance. For the post-hoc test: ^a^ *vs*. low risk. ^b^ *vs*. moderate risk. ^c^ *vs*. high risk. AVC, aortic valve calcification; ARC, aortic root calcification; BMI, Body mass index; Echo-CCS, echocardiographic calcification score; FRS, Framingham risk score; HDL-C, high density lipid cholesterol; hs-CRP, high-sensitivity C-reactive protein; KOA, knee osteoarthritis; LDL-C, low density lipid cholesterol; LVEF, left ventricle ejection fraction; MAC, mitral annulus calcification; PMC, papillary muscle calcification; SC, septum calcification; Total-C, total cholesterol; WBC, white blood cell.

Variables	Framingham Risk Score			p
	Low Risk n=79	Moderate Risk n=65	High Risk n=60	
Age, years	60.2±6.8^bc^	63.0±5.9^ac^	68.0±7.5^ab^	<0.001*
Gender, n (%)				
Female	71 (89.9)	61 (93.8)	44 (73.3)	0.003*
Male	8 (10.1)	4 (6.2)	16 (26.7)	
BMI, kg/m^2^	26.5±2.2	26.8±2.5	27.4±2.4	0.136
Smoking, n (%)	13 (16.5)	13 (20.0)	14 (23.3)	0.599
Hypertension, n (%)	20 (25.3)	44 (67.7)	54 (90.0)	<0.001*
Diabetes mellitus, n (%)	3 (3.8)	18 (27.7)	49 (81.7)	<0.001*
Grade of KOA, n (%)				
I	71 (89.9)	2 (3.1)	0	<0.001*
II	1 (1.3)	45 (69.2)	3 (5.0)	
III	5 (6.3)	16 (24.6)	27 (45.0)	
IV	2 (2.5)	2 (3.1)	30 (50.0)	
FRS	7.3 (5.3-8.6)^bc^	13.7 (12-16)^ac^	24.8 (24.1-28.5)^ab^	<0.001*
SBP, mmHg	124.5±11.3^bc^	136.5±10.9^ac^	147.6±13.7^ab^	<0.001*
DBP, mmHg	72.1±9.2^bc^	76.2±7.3^ac^	80.0±7.5^ab^	<0.001*
LVEF, %	64.4±2.7^bc^	64.3±2.3^ac^	63.2±1.9^ab^	0.006*
Echo-CCS	1 (0-1)^bc^	2 (1-3)^ac^	3 (3-4)^ab^	<0.001*
0, n (%)	35 (44.3)	7 (10.8)	0	<0.001*
1-2, n (%)	37 (46.8)	39 (60.0)	11 (18.3)	
≥3, n (%)	7 (8.9)	19 (29.2)	49 (81.7)	
PMC, n (%)	5 (6.3)	14 (21.5)	26 (43.3)	<0.001*
MAC, n (%)	10 (12.7)	15 (23.1)	40 (66.7)	<0.001*
AVC, n (%)	29 (36.7)	47 (72.3)	53 (88.3)	<0.001*
ARC, n (%)	8 (10.1)	35 (53.8)	51 (85.0)	<0.001*
SC, n (%)	12 (15.2)	6 (9.2)	23 (38.3)	<0.001*
WBC, ×10^3^/µL	8.2±2.3	8.3±2.8	8.4±1.7	0.750
Platelet, ×10^3^/µL	252.4±52.6	265.0±51.4	312.3-4±66.2^ab^	<0.001*
Glucose, mg/dL	103.0 (94.0-125.0)	113.0 (97.0-141.0)	130.0 (103.0-171.0)^ab^	<0.001*
Hemoglobin, g/dL	14.6±1.2	14.2±1.3	14.3±3.8	0.585
hs-CRP, mg/L	0.6 (0.3-0.8)^bc^	1.0 (0.8-1.5)^ac^	4.2 (2.7-5.0)^ab^	<0.001*
Total-C, mg/dL	198.0±40.1	214.8±50.0	239.0±58.4^ab^	<0.001*
HDL-C, mg/dL	54.8±11.7	54.2±10.9	50.1±7.8^ab^	<0.001*
LDL-C, mg/dL	115.1±30.1^bc^	130.0±39.8^ac^	148.8±40.1^ab^	<0.001*

## Discussion

To the best of our knowledge, this study is the first to evaluate the relationship between the severity of KOA and echo-CCS. Higher levels of CRP, echo-CCS, and FRS were associated with increase in grade of KOA. The increase in KOA grade was an independent predictor of increasing calcification scores. This was also associated with a higher CV risk.

This study both supports and extends previous population-based studies that showed that the presence or advanced grade of KOA may have a high risk of CV mortality and morbidity [6,7]. Calcification was not detected in 20.6% of patients with KOA. The rate of patients with calcification was 26.5% for one structure and 52.9% for at least two structures. It was reported that aortic tension and stretchability values were decreased, aortic stiffness and valve calcification rates were higher in KOA compared to healthy controls [5]. The presence of more cardiac calcifications can accelerate atherosclerosis and the development of CAD [9]. Echo-CCS is scored based on calcification in cardiac structures and is an essential indicator of CAD and CV events [10,23]. Previous studies have reported that echo-CCS >2 is an essential risk factor for cardiac events and all-cause mortality [9,23]. Additionally, higher echo-CCS levels have been reported in patients with KOA compared to the control group [11]. Consistent with these findings, echo-CCS levels were positively correlated with FRS and they gradually increased with the degree of KOA. Moreover, a one-degree increase in KOA increased the odds of echo-CCS 1-2 by 5.24-fold than without calcification group. Furthermore, compared to patients with an echo-CCS of 1-2, a one-grade increase in KOA was associated with a 4.96-fold increase in the odds of an echo-CCS greater than 2. These results were consistent with growing evidence that KOA may be an essential risk factor for CV diseases [3].

Traditional risk factors play an important role in the relationship between KOA and CV risk. OA data from the Framingham Heart Study reported that aortic calcification did not differ by gender [24], while the Cohort Hip and Cohort Knee (CHECK) study presented a favorable association for female gender [8]. In the current study, gender was not associated with echo-CCS. However, the female gender may be more prone to metabolic syndrome [25]. The close relationship between OA and metabolic syndrome may predispose to vascular disorders and CV diseases [26,27]. The Research on OA/Osteoporosis Against Disability (ROAD) study conducted on a population-based cohort with a three-year follow-up reported that metabolic syndrome components such as impaired glucose tolerance and hypertension are associated with the occurrence and progression of KOA [28]. Another study showed that this association persisted even after the effects of excess weight were adjusted [29]. Age, presence of hypertension, and diabetes mellitus had a positive relationship with increase in grade of KOA. However, the degree of KOA was associated with an increased calcification score, independent of traditional risk factors, particularly including age. On the other hand, Grade IV patients exhibited higher total cholesterol and LDL levels. It has been suggested that cholesterol metabolism plays an important role in OA [30], while some studies report the opposite [31,32]. Cholesterol metabolism, which plays a role in metabolic syndrome components, may be a common link for KOA [26]. Furthermore, they are involved in vascular or arterial calcification and initiation or acceleration of atherosclerosis and contribute to CV mortality [33].

Some mechanisms beyond traditional risk factors are associated with increased CV risk in KOA. Matrix Gla-protein (MGP) produced by cartilage and vessel walls is a vitamin K-dependent calcification inhibitor. Low circulating uncarboxylated MGP (ucMGP) levels may play a role in increased inflammation and accelerated atherosclerosis. It has been reported that circulating ucMGP levels are lower in both inflammatory arthritis and aortic valve disease [34,35]. In OA, a condition of inflammatory synovitis involving infiltration of macrophages and lymphocytes was associated with higher CRP levels and worse progression [36]. Z39Ig, a transmembrane protein, plays an essential role in inflammation. It is found in the human carotid artery plate and osteoarthritic synovial lining and is involved in inflammatory cell migration, cartilage destruction, and induction of chemokine or matrix metalloproteinase [37]. In addition, it has been reported that the interaction between leptin and vascular endothelial growth factor, which play an important role in CV diseases and inflammation, predisposes to the development of KOA [38]. These mechanisms support the involvement of proinflammatory cytokines and other mediators, such as nitric oxide, in the common pathways of OA and aortic valve calcifications [39,40]. Consistent with these mechanisms, a previous study has reported an independent association between systemic inflammation and aortic valve calcification [41]. The present study showed that CRP levels increased gradually with increase in grade of KOA. Furthermore, increased levels of inflammation in KOA patients were independently associated with increased calcification scores.

Limitations

This study had some significant limitations. Firstly, the sample size was partially small. Secondly, this study did not investigate proinflammatory cytokines and other mediators. A thorough evaluation of these factors might have provided greater clarity on inflammation's role, which is posited as a unifying mechanism in both KOA and intracardiac calcification pathways. Thirdly, as a confounding factor, the duration of KOA, especially in advanced-stage patients, could not be evaluated due to initial diagnoses being made at various hospitals. Finally, in a large cohort of KOA, matching the grade of KOA with traditional risk factors such as age, gender, comorbidities, family history, ethnicity, and diet could strengthen the study results.

## Conclusions

The majority of patients with KOA had intracardiac calcification. The grade in KOA was associated with FRS, a composite aggregate of CV risk factors such as hypertension and diabetes mellitus. An increased KOA grade was correlated with higher echo-CSS. These findings indicate that patients with higher grades of KOA may be predisposed to developing subclinical atherosclerosis. In patients suffering from KOA, comprehensive risk assessment with a multidisciplinary approach, including echocardiographic evaluation, can contribute to clinical management and prognosis.
